# Periprosthetic bone mineral density and fixation of the uncemented CLS stem related to different weight bearing regimes: A randomized study using DXA and RSA in 38 patients followed for 5 years

**DOI:** 10.3109/17453674.2010.487238

**Published:** 2010-05-21

**Authors:** Olof Wolf, Per Mattsson, Jan Milbrink, Sune Larsson, Hans Mallmin

**Affiliations:** Department of Orthopedics, Uppsala University Hospital, UppsalaSweden

## Abstract

**Background and purpose:**

There is no consensus on the best rehabilitation regime after uncemented total hip arthroplasty. Theoretically, bone ingrowth into the implant should benefit from initial partial weight bearing. We investigated whether the degree of postoperative weight bearing influences the periprosthetic bone mineral density (BMD) and/or the stability of the CLS stem.

**Patients and methods:**

38 patients received an uncemented CLS stem and were randomized to either unrestricted postoperative weight bearing or to partial weight bearing for 3 months. Periprosthetic BMD was measured in the 7 Gruen zones with DXA and the stability of the femoral stem was assessed by radiostereometric analysis (RSA) after surgery and at 3, 12, 24, and 60 months.

**Results:**

Periprosthetic BMD was not influenced by the type of postoperative weight bearing. BMD was reduced by 8–15% in all Gruen zones at 3 months. Restoration toward initial BMD was observed in all zones except in zone 7 (calcar region), where BMD was reduced by 22% at 5 years. Immediate weight bearing after surgery had no influence on the stability of the CLS stem, as assessed by RSA.

**Interpretation:**

Immediate full weight bearing after uncemented total hip arthroplasty is safe. There is no difference in the periprosthetic BMD or in stability of the stem as measured by RSA compared to partial weight bearing for 3 months. BMD is reduced by more than 20% in the calcar region around a CLS stem after 5 years.

The postoperative regime for uncemented hip implants has shifted towards early mobilization with full weight bearing, despite the fact that there have been discussions that early weight bearing might jeopardize the bone ingrowth that is considered necessary for stability (Soballe et al. 1993, Jasty et al. 1997). In addition, early full weight bearing might theoretically impair not only the stability of the femoral stem but also the bone mineral density (BMD) around it, but only a few studies have addressed this issue. A 2-year follow-up after total hip arthroplasty (THA) with the Bi-metric implant (Biomet Inc., Warsaw, IN) showed less bone loss proximal and lateral to the implant in a group with full weight bearing than in a group with partial weight bearing (Boden and Adolphson 2004). In 2 studies in which different uncemented femoral stems were examined, postoperative full weight bearing did not lead to any differences in micromotion over 1–2 years compared to partial weight bearing (Ström et al. 2007a, Thien et al. 2007). The roles of implant design, method of primary fixation, type of material, surface geometry, and finish are factors that may be of importance for BMD and implant stability. Thus, results for different implants should be compared with caution.

We investigated whether the amount of weight bearing immediately after implantation of an uncemented total hip arthroplasty would affect (1) the periprosthetic BMD and (2) the stability of the implant over 5 years of follow-up.

## Patients and methods

42 patients were included in this randomized controlled trial. Inclusion criteria were unilateral radiographically verified hip osteoarthritis, age between 25 and 65 years, and weight less than 110 kg. Exclusion criteria were: patients receiving steroids or other medication known to affect bone metabolism, malignancy, previous hip surgery, BMI above 35, patients living outside the Uppsala municipality. All patients gave informed consent before entering the study, which was approved by our local ethics committee (Ups 99242). Enrollment took place between April 2000 and April 2003. Patients were randomized to either unrestricted postoperative weight bearing including an intensive training program, or to partial weight bearing combined with a very restricted rehabilitation program for 3 months.

The short-term outcome has already been reported (Ström et al. 2006). 3 patients underwent contralateral THA and they have therefore been excluded from the present study. 1 patient died of pulmonary embolism 3 months postoperatively and was excluded. Thus, 38 patients (20 men) were available for the present study. The mean age of the patients was 54 (SD 9) (range 25–63) years. Their mean weight was 80 (SD 13) kg, mean height 172 (SD 9) cm, and mean body mass index (BMI) was 27 (SD 3). The distribution of women between the groups was even and the ages differed only by a few years (Table 1). The study group included some young patients and the 3 women who were younger than 40 years of age were all part of the immediate weight bearing group, as is reflected in the mean age and SD. 1 patient, randomized to partial weight bearing, had a cup revision within 20 months because of loosening, which was probably caused by manufacturing problems. This patient remained in the study for the first year after the initial surgery. 1 patient, randomized to full weight bearing, had a stem revision after 1.5 years because of aseptic loosening. This patient also remained in the study for the first year after the initial surgery. Some missing examinations meant that we could evaluate 37 patients with dual X-ray absorptiometry (DXA) and 36 patients with radiostereometric analysis (RSA) at one year. At 5 years 1 patient had died, 2 had been revised (see above), and 1 patient had moved and could not attend the follow-up examination. Of the 34 potential patients, we were able to evaluate 32 with DXA and 33 with RSA at 5 years, the omissions being due to technical problems.

**Table 1.** Patient characteristics. Values are mean (SD)

Weight bearing group	n	Age, years	Weight, kg	Height, cm
Immediate (I)				
Men	10	59 (2.6) ^**a**^	87 (9.3)	179 (5.2)
Women	8	48 (14) ^**b**^	70 (8.3)	166 (5.3)
Partial (P)				
Men	10	53 (9.6) ^**a**^	87 (11)	178 (6.0)
Women	10	55 (4.5) ^**b**^	72 (12)	163 (5.7)

^**a**^Men in P group were significantly younger than men in I group (p < 0.005).

^**b**^Women in I group were significantly younger than women in P group (p < 0.005).

The patients received an uncemented total hip replacement with the CLS hip stem (Centerpulse/ Zimmer Inc., Warsaw, IN). This is a collarless straight titanium alloy stem with a rough blasted surface, available in 13 sizes (5–20). It is a 3-dimensional taper with a trapezoidal cross-section and with anterior and posterior ribs for increased rotational stability. The design, described as a press-fit stem, allows proximal load transfer and is not thought to give any stress shielding (Spotorno et al. 1993). The original study included the uncemented Interop acetabular cup with a hemispherical porous shell with sealed screw holes. For reasons of manufacturing problems with oil-contaminated shells (Blumenfeld and Bargar 2006), the product was withdrawn from the market. Thus, only 15 Interop acetabular cups were inserted. The previously mentioned patient with the cup revision received a cup from the contaminated batch. The remaining patients were operated on with an uncemented Allofit acetabular cup without screw holes (n = 22) or an uncemented Trilogy cup with cluster holes (n = 1) (all cups by Centerpulse, Bern, Switzerland, acquired by Zimmer Inc., Warsaw, IN). The Interop cup is a press-fit cup with a modular hemispheric component with a roughened, cancellized titanium back surface, which was combined with a polyethylene hooded insert. The Allofit cup is also a press-fit cup, but with a grit-blasted titanium surface, which was combined with a polyethylene hooded insert. The Trilogy cup was combined with a polyethylene liner with a rim elevated 10 degrees. All patients received a modular 28-mm cobalt-chrome femoral head. 5 experienced surgeons performed the operations in a standardized manner in accordance with the manufacturer's manual, with an anterolateral approach.

Directly after the operation, the patients were randomized (with a closed and numbered envelope technique) to either immediate weight bearing (the I group) or partial weight bearing for 3 months (the P group). The patients in the I group were instructed to bear full weight directly after surgery, and in addition they were enrolled in a strictly controlled and active physiotherapy program with exercises to be performed at home after instruction by a specific physiotherapist. The home exercises were intensified after 4 weeks by adding training in a pool twice a week for 4–7 weeks postoperatively. After 7 weeks, intensive training in the physiotherapist's gym was added and this continued until 3 months after surgery. The patients in the P group were instructed to bear weight partially, approximately 15 kg, for 3 months, which was the routine at the department at that time. In addition, they received a short written home exercise program based on mobility through supported flexion and extension exercises. Typically, the patients in both groups were treated as in-patients for 7 days following surgery.

The proximal femur of the operated side was scanned with DXA (DPX-L; Lunar Co., Madison, WI) using the orthopedic hip implant software. The same DXA machine was used throughout the study. The baseline DXA measurement was performed during the first postoperative week. Duplicate measurements for hip implants with repositioning between the 2 scans were carried out in order to calculate the precision error (% coefficient of variance). However, at the first postoperative investigation the DXA scans were performed without repositioning in order to minimize pain in the newly operated patients. The follow-up bone densitometry measurements were carried out after 3, 12, 24, and 60 months on the same DXA machine and at those examinations duplicate measurements were performed with repositioning. Orthopedic hip implant analysis was performed for BMD (g/cm^2^) in all 7 Gruen zones, with no offset in order to include all bone outside the prosthesis. A spine phantom was scanned regularly during the study period and the long-term precision, expressed as CV% for L2-L4 BMD, was < 1.5%. The precision error was 2–4% (mean 2.8%) for the 7 zones without repositioning at the postoperative investigation and 3.2–4.2% at the other times of measurement.

The patients were evaluated through the Swedish SF-36 (Sullivan et al. 1995), a health-related quality of life rating instrument. To evaluate compliance, weight bearing was assessed at every visit to the outpatient clinic, using the F-scan system (Tekscan Inc., South Boston, MA). Mean weight bearing, expressed as peak load in kilograms, was calculated on the basis of 3 recordings, each of which included 5 steps. This was done by the same physiotherapist and with the same equipment before surgery and 1 week and 3, 6, 12, and 24 months after surgery as previously described (Ström et al. 2006).

### Hip implant DXA

BMD around the femoral stem was calculated for all 7 Gruen zones and statistical comparisons were made between the I group and the P group. The BMD for each zone was followed up longitudinally for 5 years. In addition, as there were no statistically significant differences between the 2 study groups, they were combined into one cohort to identify the CLS-specific BMD pattern. The DXA-derived mean values for the duplicate measurements were used.

### Radiostereometry

During surgery, tantalum markers were inserted into the proximal femur in a preplanned pattern. The stems were marked by the manufacturer with 5 tantalum markers, 1 at the tip, 1 on each side of the stem, and the remaining 2 in the proximal part with 1 on each side of the hump distal to the neck. Baseline RSA examinations were performed within 5–7 days postoperatively. Follow-up examinations were carried out at 1, 3, 12, 24, and 60 months. A specially trained radiology research nurse performed all examinations. A uniplanar technique was used, with the patient in the supine position, with X-ray tubes positioned at an angle of 40° with separate generators for true simultaneous exposure (Kärrholm et al. 1997, Valstar et al. 2005). The precision was based on 42 duplicate examinations and a 95% confidence interval was chosen as the limit for significant motion (Table 2). Migration of the stem was measured as rotations around 3 axes and as translations of the center of the rigid body along these 3 axes. Examinations with a scatter corresponding to a condition number (CN) of < 120 and a stability corresponding to < 0.3 mm (mean error of rigid body fitting, ME) were included. UmRSA Analysis software version 6.0 (RSA Biomedical, Umeå, Sweden) was used for measurements and calculations.

**Table 2.** Precision of stem migration

Type of motion	Stem
Translation (mm)	
Transverse axis (x)	0.12
Longitudinal axis (y)	0.11
Sagittal axis (z)	0.31
Rotation (degrees)	
Transverse axis (x)	0.42
Longitudinal axis (y)	1.06
Sagittal axis (z)	0.12

95% prediction interval about zero for significant motions based on 42 double examinations.

### Statistics

Mean DXA values in the different Gruen zones were compared between groups with an unpaired t-test, while RSA translations and rotations were compared with the Mann-Whitney U test, since the values were not normally distributed. Statistical analysis was performed with Statistica software version 8.0 (StatSoft Inc, Tulsa, OK). Differences were considered significant when p < 0.05. Values are given as mean (SD). SF-36 dimensions were only viewed in graphs and were not compared statistically. Before the study, a power analysis was done to estimate the sample size necessary to detect a significant difference in linear micromotion along the y-axis (i.e. subsidence) between the 2 groups with alpha = 0.05 and beta = 0.20 (i.e. a power of 0.8), and groups of 20 patients were found to be sufficient.

## Results

### Bone mineral density

**Figure 1.**
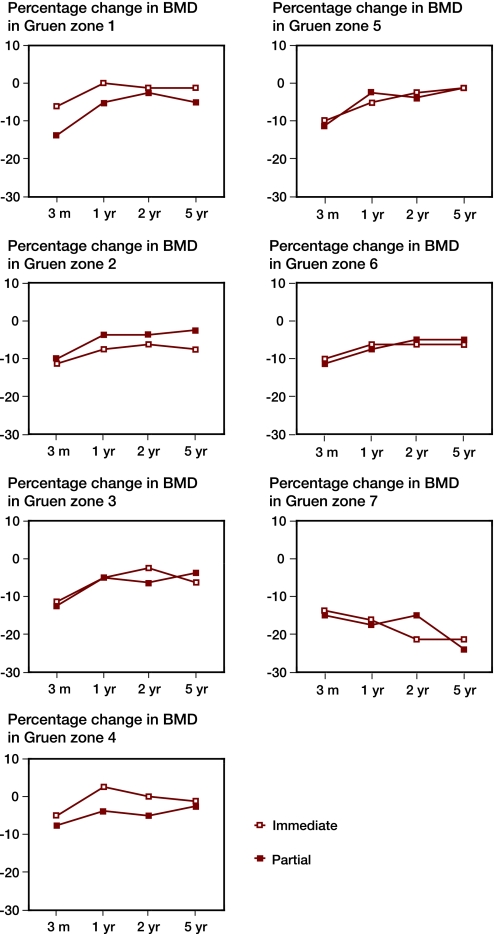
Percentage change in BMD in different Gruen zones. Immediate weight bearing group compared to partial weight bearing group. Changes from baseline (at one week) to 3 months, 1, 2, and 5 years.

**Figure 2.**
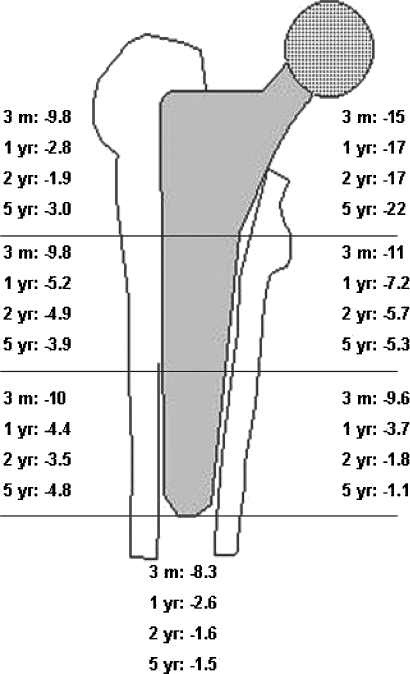
Mean percentage change in BMD in different Gruen zones in the 2 study groups combined. Changes from baseline (at 1 week) to 3 months, 1, 2, and 5 years.

There were no statistically significant differences in BMD in any of the Gruen zones at any measurement time between the study groups (Table 3, see Supplementary data, and Figure 1). When in an additional analysis the groups were combined and assessed as one group with regard to the different Gruen zones, an 8–15% decrease in BMD was found in all zones at 3 months. This was followed by restoration of BMD toward the baselines in all zones except zone 7. For zone 7 (i.e. the proximal medial zone) BMD decreased progressively, reaching –22 % at 5 years (Figure 2).

### Stability of the femoral stem

The only difference between the groups occurred at 3 months, as a difference in translation along the z-axis, with anterior translation of the center of the rigid body in the I group and posterior translation in the P group (Table 4, see Supplementary data). There was no significant difference in subsidence of the stem, i.e. translation along the y-axis, between groups; nor was there any significant difference in anteversion or retroversion, i.e. rotation around the y-axis, between groups. At 5 years, the mean subsidence was 1.7 (2.7) mm, mean retroversion 3.0 (8.2) degrees and mean varus tilt 0.4 (1.1) degrees. The other micromotions were within precision limits at 5 years.

The patient who was revised at 1.5 years showed subsidence of the stem of 1.5 mm at 3 months and 3.4 mm at 1 year. There was no movement in retroversion at 3 months, but 13 degrees of retroversion was seen at 1 year. 5 patients (2 in the I group and 3 in the P group) had a subsidence greater than that in the revised patient at 1 year (3.5–6.4 mm), although there was less than 5 degrees retroversion and they were all asymptomatic. These stems remained stable at 5 years. At the 5-year follow-up, another patient (I group) had subsidence of 10 mm and retroversion of 45 degrees. At the 1- and 2-year examinations, subsidence was 0.3 and 1.0 mm and retroversion was 1.2 and 7 degrees, respectively.

### SF-36

There were no differences in any of the SF-36 dimensions between the groups before surgery, at 3 months, and at 1 and 2 years after surgery. Before surgery, the scores for general health (GH) and mental health (MH) in the study groups were similar to those in the Swedish age-matched reference population, but the individuals in the study groups generally had lower scores for all other dimensions. At 3 months, physical functioning (PF) and role limitations due to physical functioning (RP) were still much lower in the study groups, whereas the means for the other dimensions were equal to those of the reference population. At 1 and 2 years, all dimensions of SF-36 were equal to those of the reference population.

### Weight bearing

Patients in the I group put a greater load on the operated leg at 1 week and at 3 months than patients in the P group. The P group did comply to given instructions to some extent, but loaded approximately 30 kg on the operated leg during the first 3 months.

## Discussion

Full weight bearing immediately after operation did not show any negative effects on the primary outcomes, implant stability and BMD, around an uncemented femoral stem. In addition, the SF-36 scores (Sullivan et al. 1995) were not affected by differences in postoperative regimes.

BMD changes around cemented and uncemented primary THAs have been described in several previous reports (Venesmaa et al. 2001, 2003, Aldinger et al. 2003, Boden and Adolphson 2004, Boden et al. 2006, Albanese et al. 2009). In a combined DXA and RSA study of 5 different cemented implants, no association between stem migration and bone mineral density changes was seen after 5 years (Li et al. 2007).

The pattern of BMD changes with different uncemented femoral stems in primary THA varies. Theoretically, it is mainly a matter of stem design and more specifically an effect of where the femoral stem is fixed—and thereby where stress is created on the surrounding bone. For instance, distally fixated stems will lose bone proximally as a result of stress shielding. This was illustrated in a comparison of the ABG I and II (Van der Wal et al. 2006). Some of the studies with moderate or long-term follow-up have had a cross-sectional design (Niinimaki et al. 2001, Aldinger et al. 2003, Sköldenberg et al. 2006, Albanese et al. 2009). There have been few longitudinal BMD studies with a follow-up of 5 years or more; those that exist involved the hydroxyapatite coated Bi-metric stem (Boden et al. 2006), the press fit titanium Spotorno stem (Aldinger et al. 2003), the Zwey-Muller, the Corail, the Optifix, and the Autophor 900S (Karachalios et al. 2004), and the cemented Lubinus SP II (Venesmaa et al. 2003). In several of these studies, a decrease in BMD occurred during the first 1 or 2 years in all zones. Often, there is a successive catch up of BMD in the distal zones, but ongoing bone loss in the calcar region (zone 1 or 7, or both) depending on the type of stem. Bodén and Adolphson (2004) found a progressive loss of bone in zones 1 and 7 after 2 years in 20 patients with the Bi-metric stem. However, only a few studies have been designed as randomized controlled trials, and to our knowledge only 1 study has focused on the issue of differences in postoperative weight bearing and the effect on BMD around an uncemented stem. Bodén and Adolphson (2004) found that in zones 1, 4, and 5 there was more bone loss in their P group at 3 months, but at 2 years BMD was only lower in zone 1. This contrasts with our finding that there were no differences between the groups at any time point. Moreover, while we observed restoration of BMD in all zones except zone 7, they found no restoration in zones 1 or 7. These differences might be the result of different stem designs. Another study of long-term BMD results in 14 patients with the Bi-metric stem showed progressive reduction in the proximal zones after 6 and 14 years (Boden et al. 2006). The bone mineral density around the titanium-blasted press-fit CLS stem in our study was unaffected by the postoperative regime. When the 2 groups were assessed as 1 combined group, a statistically significant reduction was found only for the medial proximal zone, approximately in the calcar region, after 5 years. We used the first postoperative examination as baseline for DXA measurements, as previously described (Venesmaa et al. 2001). In contrast to several other studies, we followed BMD only in the operated hip, and not by an overlay to the healthy hip. Any effects of aging on BMD were not, therefore, taken into account, which could be of some importance. Women with low systemic BMD show greater bone loss in the calcar region after uncemented THA than women with normal BMD (Alm et al. 2009). In our study, the distribution of women between the two groups was even and the ages differed only by a few years (Table 1).

To our knowledge, only 2 studies (Ström et al. 2007b, Thien et al. 2007) have addressed the question of postoperative weight bearing and implant stability with use of RSA to measure micromotion. Thien et al. (2007) published 1-year results and Ström et al. (2007b) published 2-year results and found no difference between patients who had been recommended immediate full weight bearing and those recommended partial weight bearing. We found a difference only in anterior and posterior translation. However, the mean translation in both groups was small: approximately 0.1 mm. This is well within the precision limits of the RSA method in this setting; hence, no true motion might have occurred.

Our patients who were randomized to partial weight bearing were instructed by a physiotherapist to load a maximum of 15 kg on the operated leg. Compliance with the weight bearing instruction was measured at the visits to the physiotherapist, using the F-scan system. The patients in the P group put almost twice the recommended weight on the operated side. They did, however, put less load on the operated leg at 1 week and 3 months than the I group. Others have used an auditory device calibrated to between 10% of body weight to 30 kg of loading to instruct the patients (Boden and Adolphson 2004, Thien et al. 2007).

Our study has some limitations. The sample size was determined from calculations of the power required to detect micromotions of an uncemented femoral stem by RSA, and thus not to detect differences in BMD. On the other hand, our study had a longer follow-up (5 years as opposed to 2 years) and was almost twice as large as the only similar study, which actually showed differences (Boden and Adolphson 2004). As previously mentioned, the analysis of BMD did not consider sex and aging as factors associated with BMD. This would probably not have affected the comparison between the I and P groups, since the female to male distribution was equal and age differences were small. However, whether the sex- and age-related generalized decrease in BMD also affects the skeleton around a femoral stem implant, cemented or not, is at present unknown.

Our study also has certain merits. The use of an RCT design contributes to the ability to draw valid conclusions. We acknowledge that there was a small sample size, but by calculating the mean value from duplicate DXA measurements we increased the precision of measurement of BMD. The compliance with the postoperative weight bearing regimes was checked by gait analysis.

## Supplementary data

Supplementary Tables 3 and 4 are available at our website (www.actaorthop.org), identification number 3888/10.
