# Supplementary material for: Periprosthetic bone mineral density and fixation of the uncemented CLS stem related to different weight bearing regimes: A randomized study using DXA and RSA in 38 patients followed for 5 years
Source: Acta Orthop. 2010 May 21;81(3):286–91. doi: 10.3109/17453674.2010.487238 (PMC2876828; doi:10.3109/17453674.2010.487238)
Supplement: Supplementary file 1 [file ORT-1745-3674-81-286-s1.doc]

Table 3. Bone mineral density (BMD) and percentage change in BMD in the 7 different Gruen zones around the CLS femoral stem up to 5 years after surgery. Immediate postoperative weight bearing versus partial weight bearing.

Gruen zone Exam. IWB n %Δpo PWB n %Δpo p

1 po 1.02 (0.2) 18 1.01 (0.2) 20 0.9

3m 0.97 (0.2) 17 -5.7 0.88 (0.2) 20 -13.4 0.2

1y 1.02 (0.2) 18 0.3 0.96 (0.2) 19 -5.8 0.3

2y 1.00 (0.2) 15 -0.9 0.98 (0.2) 19 -2.7 0.8

5y 1.02 (0.2) 14 0.2 0.97 (0.3) 18 -5.5 0.6

2 po 1.96 (0.2) 18 1.98 (0.2) 20 0.7

3m 1.78 (0.2) 17 -10.2 1.80 (0.2) 20 -9.5 0.8

1y 1.83 (0.2) 18 -6.6 1.90 (0.2) 19 -4.0 0.3

2y 1.84 (0.2) 15 -6.2 1.89 (0.2) 19 -3.9 0.4

5y 1.87 (0.3) 14 -5.7 1.92 (0.3) 18 -2.5 0.6

3 po 2.14 (0.2) 18 2.15 (0.2) 20 0.8

3m 1.94 (0.2) 17 -9.7 1.93 (0.2) 20 -10.6 0.9

1y 2.03 (0.2) 18 -4.9 2.06 (0.2) 19 -4.0 0.7

2y 2.08 (0.2) 15 -3.0 2.07 (0.2) 19 -3.9 0.9

5y 2.01 (0.4) 14 -6.2 2.07 (0.2) 18 -3.7 0.6

4 po 1.96 (0.2) 18 1.99 (0.2) 20 0.7

3m 1.80 (0.2) 17 -8.2 1.82 (0.2) 20 -8.3 0.7

1y 1.93 (0.2) 18 -1.2 1.92 (0.3) 19 -3.8 0.8

2y 1.96 (0.1) 15 0 1.92 (0.3) 19 -2.8 0.6

5y 1.96 (0.2) 14 -0.6 1.94 (0.3) 18 -2.2 0.8

5 po 2.16 (0.2) 18 2.17 (0.2) 20 0.9

3m 1.93 (0.2) 17 -10.5 1.98 (0.2) 20 -8.8 0.5

1y 2.05 (0.2) 18 -5.3 2.11 (0.2) 19 -2.2 0.3

2y 2.10 (0.2) 15 -2.3 2.13 (0.2) 19 -1.4 0.6

5y 2.13 (0.2) 14 -1.3 2.14 (0.2) 18 -1.0 0.9

6 po 1.81 (0.3) 18 1.82 (0.2) 20 0.9

3m 1.62 (0.3) 17 -10.5 1.60 (0.2) 20 -11.5 0.9

1y 1.67 (0.3) 18 -7.1 1.69 (0.2) 19 -7.3 0.9

2y 1.64 (0.3) 15 -6.2 1.71 (0.2) 19 -5.3 0.5

5y 1.71 (0.3) 14 -5.2 1.71 (0.3) 18 -5.3 1.0

7 po 1.26 (0.2) 18 1.26 (0.2) 20 1.0

3m 1.07 (0.2) 17 -14.8 1.07 (0.2) 20 -15.0 1.0

1y 1.05 (0.2) 18 -17.0 1.05 (0.2) 19 -17.5 1.0

2y 0.98 (0.2) 15 -20.8 1.08 0.2) 19 -14.6 0.2

5y 1.00 (0.2) 14 -19.5 0.97 (0.2) 18 -23.7 0.7

Examination po= postoperative within 7 days; 3 months; 1, 2, and 5 years after surgery. IWB= immediate weight bearing. PWB= partial weight bearing. n = number of patients. %Δpo = percentage change from first postoperative examination. p = p value, t-test.
