# Supplementary material for: Periprosthetic bone mineral density and fixation of the uncemented CLS stem related to different weight bearing regimes: A randomized study using DXA and RSA in 38 patients followed for 5 years
Source: Acta Orthop. 2010 May 21;81(3):286–91. doi: 10.3109/17453674.2010.487238 (PMC2876828; doi:10.3109/17453674.2010.487238)
Supplement: Supplementary file 2 [file ORT-1745-3674-81-286-s2.doc]

Table 4. Micromotion of the CLS femoral stem along and around the three axes measured with RSA up to 5 years after surgery. IWB versus PWB.

IWB PWB

Mean (SD) Mean (SD) p

1 month (n=18/20)

X-translation, mm, medial+/lateral- -0.01 (0.1) 0.02 (0.1) 0.6

Y-translation, mm, proximal+/distal- -0.38 (1.0) -0.42 (0.9) 0.3

Z-translation, mm, anterior+/posterior- 0.02 (0.1) -0.09 (0.2) 0.06

X-rotation (°), anterior+/posterior- tilt -0.14 (0.3) -0.12 (0.2) 0.8

Y-rotation (°), retroversion+/anteversion- 0.55 (0.8) 0.69 (1.4) 0.5

Z-rotation (°), valgus+/varus- tilt -0.04 (0.1) -0.05 (0.2) 0.7

3 months (n=18/19)

X-translation, mm, medial+/lateral- -0.04 (0.2) 0.04 (0.2) 0.2

Y-translation, mm, proximal+/distal- -1.00 (1.7) -1.01 (1.7) 0.6

Z-translation, mm, anterior+/posterior- 0.15 (0.2) -0.09 (0.2) 0.002

X-rotation (°), anterior+/posterior- tilt -0.15 (0.3) -0.10 (0.3) 0.4

Y-rotation (°), retroversion+/anteversion- 0.64 (1.0) 0.92 (1.6) 0.9

Z-rotation (°), valgus+/varus- tilt 0.00 (0.2) -0.08 (0.2) 0.2

1 year (n=17/19)

X-translation, mm, medial+/lateral- 0.03 (0.3) 0.00 (0.2) 0.6

Y-translation, mm, proximal+/distal- -1.42 (2.1) -1.03 (1.8) 0.09

Z-translation, mm, anterior+/posterior- -0.01 (0.3) -0.06 (0.3) 0.7

X-rotation (°), anterior+/posterior- tilt -0.02 (0.7) 0.00 (0.3) 0.4

Y-rotation (°), retroversion+/anteversion- 1.83 (3.0) 1.04 (1.6) 0.2

Z-rotation (°), valgus+/varus- tilt -0.15 (0.6) -0.14 (0.2) 0.9

2 years (n=17/19)

X-translation, mm, medial+/lateral- 0.05 (0.3) 0.01 (0.2) 0.6

Y-translation, mm, proximal+/distal- -1.42 (2.1) 1.25 (1.9) 0.3

Z-translation, mm, anterior+/posterior- -0.10 (0.7) -0.12 (0.3) 0.2

X-rotation (°), anterior+/posterior- tilt -0.19 (0.7) 0.04 (0.4) 0.4

Y-rotation (°), retroversion+/anteversion- 2.39 (3.7) 1.02 (1.6) 0.2

Z-rotation (°), valgus+/varus- tilt -0.16 (0.6) -0.07 (0.3) 0.5

5 years (n=16/17)

X-translation, mm, medial+/lateral- -0.11 (0.5) 0.07 (0.3) 0.4

Y-translation, mm, proximal+/distal- -2.13 (3.2) 1.33 (2.1) 0.1

Z-translation, mm, anterior+/posterior- -0.36 (1.3) -0.13 (0.3) 0.5

X-rotation (°), anterior+/posterior- tilt 0.36 (1.4) 0.23 (0.8) 1.0

Y-rotation (°), retroversion+/anteversion- 4.90 (11.6) 1.26 (1.8) 0.1

Z-rotation (°), valgus+/varus- tilt -0.62 (1.5) -0.16 (0.3) 0.3

IWB= Immediate weight bearing group. PWB= partial weight bearing group. n= number of patients in IWB/PWB group. p= p value, Mann-Whitney U test.
